# To Be (Vaccinated) or Not to Be: The Effect of Media Exposure, Institutional Trust, and Incentives on Attitudes toward COVID-19 Vaccination

**DOI:** 10.3390/ijerph182412894

**Published:** 2021-12-07

**Authors:** Dorit Zimand-Sheiner, Ofrit Kol, Smadar Frydman, Shalom Levy

**Affiliations:** 1 School of Communication, Ariel University, Ariel 40700, Israel; ofritk@ariel.ac.il; 2 Doctoral School, Jaume I University, 12071 Castelló de la Plana, Spain; darifry@gmail.com; 3 Department of Economics and Business Administration, Ariel University, Ariel 40700, Israel; shalom@ariel.ac.il

**Keywords:** COVID-19, pandemic, vaccination, media trust, institutional trust, incentives

## Abstract

The COVID-19 vaccine has become a strategic vehicle for reducing the spread of the pandemic. However, the uptake of the vaccine by the public is more complicated than simply making it available. Based on social learning theory, this study examines the role of communication sources and institutional trust as barriers and incentives as motivators of people’s attitudes toward vaccination and actual vaccination. Data were collected via an online panel survey among Israelis aged 18–55 and then analyzed using structural equation modeling (SEM). Findings show that social media trust negatively mediates the effect of exposure to information on the vaccine on attitudes toward vaccination. However, mass media trust and institutional trust positively mediate this relationship. Incentives were effective motivators for forming positive attitudes and moderating the effect of institutional trust on attitude toward vaccination. This study facilitates a deeper understanding of health communication theory in pandemics and makes important recommendations for practitioners and policy makers.

## 1. Introduction

In December 2020, the American FDA approved the first COVID-19 vaccine, and it has become a major strategic vehicle for diminishing the spread of the pandemic. The clear expectation of widespread global vaccination was premised on the assumption that the public would be willing to receive it if offered. However, this premise was shown to be unfounded [1], and a public health battle emerged between governments and anti-vaccination movements [2]. The public was confronted by misguided information and fake news, mainly through social media, which undermined their decision making and increased their hesitation regarding the vaccine. Consequently, COVID-19 vaccine uptake promotion campaigns were inevitable [2]. The confrontation with COVID-19 vaccine refusals intensified the importance of understanding the barriers and motivators of public vaccination and how formal and informal institutions can effectively communicate with the public about it [1]. Credible communication can reach a collective consciousness and establish attitudes and beliefs that can affect the willingness to receive the vaccine [3].

The current study aims to focus on communication barriers such as trust in communication sources and governmental institutions and motivators such as government incentives and examine their role in enhancing public willingness to be vaccinated during times of pandemic. Although there is extensive research regarding individual motivation, attitude, and concern for vaccination acceptance and refusal [4,5], the unique context of the rapid development of the COVID-19 vaccination, the worldwide pandemic, and the varied global political realities might result in different findings [6]. Additionally, recent studies in health communication regarding the COVID-19 pandemic have focused on individual health beliefs [7], individual personality [8], and perspectives or religious beliefs [9]. However, although there is a need for a deeper understanding of the effect of individual exposure to information about vaccination and what motivates or discourages a favorable decision to be vaccinated, the research on these subjects is still scarce. Therefore, based on social learning theory [10], the current study offers a conceptual framework that integrates exposure to information on vaccination with key mediating factors—believability of information sources and institutional trust—to explain attitudes toward vaccination and actual vaccination.

The contribution of the current research lies in offering a unique conceptual framework contributing to health communication literature. Specifically, it highlights the effect of trust and incentives on the learning process and consequent behavior, corresponding with the social learning theory and the health belief model. Moreover, this framework further contributes to governments and policy makers who need to have more understanding of how to communicate effectively with the public and convince them to be vaccinated during times of pandemic, overcoming the misinformation in the media and individuals’ misinformed beliefs.

### 1.1. Social Learning Theory and Exposure to Vaccine Information

During a pandemic, public exposure to health communication disseminated from diverse sources forms attitudes toward the communicated message and consequential behavior [11]. The response to intimidating information about the pandemic and recommendations for preventive actions highly depends on individuals’ exposure to trustworthy information sources [12]. To theoretically frame this perspective, the current study applies social learning theory (also referred to as social cognitive theory) [10,13,14], a well-established theory derived from psychology and education research [15,16,17]. It was later extended to media research dealing with the effect of media on individuals and is also part of the health belief model used to explain the adoption of preventative health behaviors [18]. Social learning theory asserts that human behavior is governed by a triadic determinism: social environment, cognitive abilities, and behavior. These three factors maintain an ongoing interaction and affect one another. The primary source of information is the social environment, including media messages, upon which the individual establishes his cognitive perceptions of the world, beliefs, expectations, and self-perception.

Next, a person uses his cognitive abilities to process the inputs he receives from the environment, develop expectations, and decide how to act. His behavior affects the environment and changes it. The changed environment provides new information to the cognitive system. Hence, social learning theory employs a basic approach that media exposure plays a vital role in social learning, demonstrating the effect of exposure to media on attitude formation and behavior. Previous research presented the relation between social learning and visual culture [19], and the effect of social learning in social commerce sites, forums, and online communities on consumer decision making and purchase intentions [20]. In the context of a pandemic, research demonstrated how exposure to information in mass media plays an essential role in addressing misinformation and changing beliefs about the source, modes of infection and prevention of the Ebola pandemic [15]. Based on this theory, it is suggested that people’s exposure to the social environment formed by traditional and social media (distributing information about the vaccine) will affect individuals’ cognitive perceptions manifested in their attitude toward vaccination. Accordingly, a positive attitude toward vaccination will be followed by a decision to act. Therefore, the following hypotheses:

**Hypothesis** **1 (H1).** *Level of exposure to media communication regarding the COVID-19 vaccination directly affects attitudes toward vaccination.*

**Hypothesis** **2 (H2).** *Attitude toward COVID-19 vaccination is positively related to actual vaccination.*

Social learning theory and the stimulus–organism–response (SOR) paradigm posit that the effect of environmental cues (the stimuli), such as exposure to information about the COVID-19 vaccine in the media on individual attitudes and behavior, is mediated by the individual’s (organism) internal state, including cognitive judgments and affective response aroused by environmental cues. These perspectives suggest that the response to exposure to information from media sources is also affected by evaluation of the media environment. Qiao et al. [21] found that while consumers use multiple sources for information about the COVID-19 vaccine, their vaccine acceptance is dependent on the level of trust in these information sources. Additionally, research indicates a possible change in trust generated by media exposure. For example, exposure to specific media content may cause affective disposition toward the content which creates an indirect positive effect on media trust [22], while consumption of misinformation is associated with a general decrease in media trust [23]. Bearing in mind the above discussion, we consider trust as an internal state of the organism (the individual) which is activated during exposure to information from media sources.

### 1.2. Trust in Information Sources and Attitude toward Vaccination

Trust has been an essential factor between two parties who wish to exchange values and ideas. The success of the exchange process builds trust and supports the relationship between the main actors [24], and is a vital element for the optimal function of the society [25]. Previous research has shown that trust in information sources is an important aspect of information-seeking behavior during crises [26,27]. Individuals’ trust in the institutions providing the information, such as media institutions and government agencies, affects their vaccination intentions and behavior [27].

#### 1.2.1. Trust in the Media

People are exposed to information about COVID-19 vaccination from two primary sources: mass media sources (such as news on T.V., newspapers, and radio) and social networks, incorporating information created by people we know or follow online [7,28]. Previous research has demonstrated that an individual’s trust and credibility perception vary by information source [28,29,30].

Trust is defined as one’s confidence in the information channels to provide accurate, fair, trustworthy, and unbiased information [22]. This perception of trusted sources delivering information is an essential aspect of people’s willingness to adopt the information [31]. Thus, vaccination attitude might be affected by the trust in the validity of the information provided by the government and the media, which largely disseminate this information [27].

#### 1.2.2. Social Media Sources and Trust

Social media sources such as Facebook and Twitter play a significant role in facilitating the exchange of relevant information during an epidemic [32,33]. The widespread public adoption of social media platforms as a tool for information seeking has led to a flood of misinformation about COVID-19 and false narratives relating to the vaccine [1,34]. Moreover, conspiracy theories have flourished due to the lack of censorship on social media platforms [35]. However, recommendations from family and friends on social media platforms may carry more weight than those from government officials or other spokespeople. Social media plays an important role in vaccination decision making [1]. Individuals tend to trust the accuracy of information when it comes from others with whom they perceive to share similar interests [36,37]. Hence, individuals’ shared interests on social media may function as a heuristic for trustworthiness [36], which contributes to the persuasive effect of the communication [38].

Previous research demonstrates that information originating on social media sites and created by other users affects individuals’ attitudes toward vaccination behaviors. These attitudes can lead to a pro-vaccination stance and are driven by the receiver perceptions of information credibility [34,38]. Moreover, Turcotte et al. [39] conclude that “Social recommendations from people perceived as quality opinion leaders led to an increase in outlet trust… These results extended beyond trusting a news outlet to indicators of future behavior”. This implies that trust further mediates the effect of exposure on attitude and behavior. Therefore

**Hypothesis** **3a (H3a).** *Social media trust positively affects attitudes toward vaccination.*

**Hypothesis** **3b (H3b).** *The relationship between the exposure to vaccine information and attitude toward vaccination is mediated by social media trust.*

#### 1.2.3. Mass Media and Trust

Mass media is an essential source of information and still plays a significant role in influencing health-related outcomes [40]. A recent study revealed that most U.S. citizens (86%) use mass media to obtain information on the COVID-19 vaccine, as these sources are perceived as high-quality for sharing fact-based vaccine information linked to governmental, healthcare, or academic data and reports [27,28]. Moreover, obtaining information from mass media increased vaccination acceptance [28].

Trust is considered a crucial variable for mass media effects [41]. Prior research has revealed that people’s trust in mass media affects their tendency to follow preventive health suggestions from this source [27]. Niu et al. [7] found that T.V. trust was significantly associated with preventive behaviors during the COVID-19 outbreak. Moreover, individuals’ attitudes toward vaccination were related to their trust in the accuracy of information provided by the media [27]. The importance of media trust was particularly notable concerning vaccination attitudes. Recent research on college students found that although they were exposed to variety of media, the level of trust in these media sources influenced their vaccine acceptance [21]. These studies further suggest that after media exposure there is a possible effect of information evaluation (i.e., trust) on attitude formation. Hence, our hypotheses:

**Hypothesis** **4a (H4a).** *Mass media trust positively affects attitude toward vaccination.*

**Hypothesis** **4b (H4b).** *The relationship between the exposure to vaccine information and attitude toward vaccination is mediated by mass media trust.*

#### 1.2.4. Institutional Trust

Institutional trust is used to describe citizens’ trust in actors such as governmental organizations [25]. It was found that institutional trust is a distal factor influencing individuals’ vaccination hesitancy and is part of evolving conspiracy theories emphasizing distrust of government organizations [4,34,42].

Institutional trust is an underlying factor in the effectiveness of the democratic process. It is based on citizens’ prior experience and familiarity with information about these institutions’ fair (or unfair) conduct [43]. Thus, institutional trust is based on prior knowledge about the trustworthiness of the concerned institutions. Furthermore, studies on institutional trust claim that media exposure is related to institutional trust since most of the information about the past behavior of governmental institutions originates from media sources [44]. Moreover, research on exposure to vaccine-related information reveals that while trust in information about the vaccine is positively related to attitudes and behaviors, it is not enough when associated institutions are mistrusted [45,46].

Additionally, previous research demonstrates that mistrust is a more common reason for negative attitudes toward vaccination than lack of information [47]. For instance, Vinck et al. [48] found that low institutional trust was highly associated with negative attitudes toward acceptance of Ebola vaccines, while Borah and Hwang (2021) found that trust in doctors’ vaccine recommendations positively mediates between doctor-patient communication and vaccination attitudes [47]. Thus, our hypotheses are:

**Hypothesis** **5a (H5a).** *Institutional trust positively affects attitudes toward vaccination.*

**Hypothesis** **5b (H5b).** *The relationship between the exposure to vaccine information and attitude toward vaccination is mediated by institutional trust.*

### 1.3. Incentives

Incentives are defined as “any action taken by the authorities that may lead to an increase in the level of vaccination coverage” [48]. This implies that there is a need for a change in citizen attitudes toward the vaccine and/or toward the action of being vaccinated which may be changed by offering some encouragement. For instance, where citizens’ hesitancy about the COVID-19 vaccine was found to inhibit the uptake of COVID-19 vaccination in the Israeli population, the government and local authorities started offering various incentives. The incentives varied from small gifts to granting a ‘green pass’, which allows citizens to participate in cultural events, eat in restaurants, stay at hotels, etc. [49]. The vaccine was also made accessible in town centers and public buildings rather than in health centers [50].

Previous research indicates that incentives affect consumer health care choices. Specifically, it was found that incentives raise the level of citizens’ consent to be vaccinated [51,52]. Thus:

**Hypothesis** **6 (H6).** *Incentives positively affects attitude toward vaccination.*

It has already been shown that coercive policies, which are the opposite of incentives, can damage trust in vaccination operations, while incentives may help in promoting vaccinations when trust in institutions is low [53,54]. While coercive policies are perceived as an ‘illiberal’ approach that strengthens the vaccination resistance, incentives create the notion of autonomy and voluntariness [55]. Recent research performed among American and Canadian adults during the COVID-19 pandemic found that respondents with a negative attitude toward being vaccinated were more affected by information about the vaccine safety as an incentive (38%) than financial incentives (18%), permission to attend the workplace (31%), coupons or discounts (8%) or tickets to sports events (19%) [56]. Though these respondents are motivated mainly by mistrust in the institutions promoting the vaccine, other incentives motivate individuals with low institutional trust to get vaccinated. In other words, the incentive becomes a motivating factor for a positive attitude toward vaccination in cases where there is less trust in the institutions that promote and support the vaccine.

**Hypothesis** **7 (H7).** *Incentives negatively moderate the effect between institutional trust and attitude toward vaccination.*

**Figure 1:**
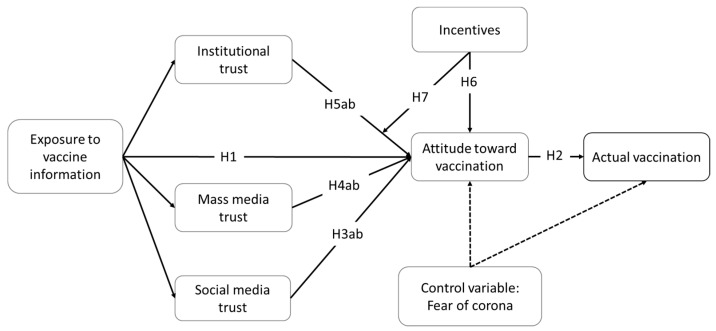
The conceptual framework.

The conceptual framework is shown in Figure 1.

## 2. Materials and Methods

### 2.1. Data Collection and Sample

To test the hypotheses empirically, data were collected via a self-administered questionnaire. Israeli participants were randomly recruited via an online panel survey company, Blueberries. The university’s ethics committee where this study was conducted has confirmed that this study meets the conditions set out in the procedure for approving a study that is not a clinical trial in humans. All respondents were assured of confidentiality, and consent was obtained from all participants before the questionnaire. The questionnaires were coded for anonymous data analysis.

The unique advanced stage of Israel’s vaccination situation made Israel a valuable case study for vaccination behavior and attitudes. At the time of data collection, a high percentage of Israelis were already vaccinated against COVID-19 (especially among high-risk groups), the vaccine supply was higher than the demand, and Israel authorities were highly active with an extensive vaccination media campaign. The chosen population was restricted to adults of age 18–55. The group of people age 55+ were excluded since the majority of them were already vaccinated and contribute less to the research purpose.

Overall, 863 respondents entered the survey. Respondents who did not belong to the chosen population were screened out at the beginning of the questionnaire and did not participate in the survey. The final sample included 484 usable responses. In this sample, participants were 57% females and 43% males. The age of participants ranged from 18 to 55 years (M = 36.6, SD = 10.6). Most of the participants had an average or below-average income (69%) and post-secondary education (76%). Among them, 41% were vaccinated against the COVID-19, 19% had natural immunity due to COVID-19 infection, and 5% stated that a physician prohibited their vaccination.

### 2.2. Variable Measurement

The survey questionnaire scales consisted of items primarily gathered from previously validated studies, while a few specific new ones were designed for the current study (see Table 1). Where necessary, the scale items were modified to capture vaccination orientation. Actual vaccination was measured by a single item expressing participants’ actual vaccination. In this measure, respondents were asked to answer yes/no to the question: “I was vaccinated against COVID-19 at least once”. Items for attitude toward vaccination were taken from Fu et al. [57]. Mass media trust and Social media scales were taken from Peifer [22]. Institutional trust items are original and adjusted for the current study based on Ervasti et al. [25]. Similarly, the items for effect of incentives are original and designed for the current study. Here, respondents were asked to indicate their level of agreement with different statements, on a seven-point Likert scale, ranging from 1 = strongly disagree, to 7 = strongly agree. Items for exposure to vaccine information are adjusted for this study and based on Venkatesh et al. [58] and Kol et al.’s [31] orientation. Respondents were asked to indicate their level of exposure to different information sources on a seven-point Likert scale, ranging from 1 = not exposed at all, to 7 = highly exposed.

An additional variable was added as a control variable. Fear of pandemic was added to control for the extrinsic effect of fear during the pandemic, which can be an excuse for the people’s attitude toward vaccination and actual vaccination. The scale was adopted from Tran [59]. Here, respondents were asked to indicate their level of agreement with different statements on a seven-point Likert scale, ranging from 1 = strongly disagree, to 7 = strongly agree. Demographic variables were also gathered.

## 3. Results

### 3.1. Validity and Reliability

First, to ensure that no Common Method Bias (CMB) exists in variance, Harman’s one-factor test was used, and a single factor accounted for just 31.77 of the (total) variance, indicated that bias is no serious concern of CMB.

Next, for validity concern, a confirmatory factor analysis (CFA) was executed. An acceptable fit was found in all measurements (χ^2^ value (319) = 778.08, *p* < 0.001 (χ^2^/df < 3); Comparative Fit Index (CFI) = 0.956; Normed Fit Index (NFI) = 0.929; and Root Mean Square Error of Approximation (RMSEA) = 0.055). The standardized regression estimate for each factor was above 0.50, displaying acceptable fit of the measures. Table 1 displays the convergent validity and reliability measures (average variance extracted (AVE), composite reliability (CR) and Cronbach’s alpha) and Table 2 presents correlation pattern between variables and the maximum shared squared variance (MSV) indicating discriminant validity of all constructs.

### 3.2. Empirical Findings

**Figure 2:**
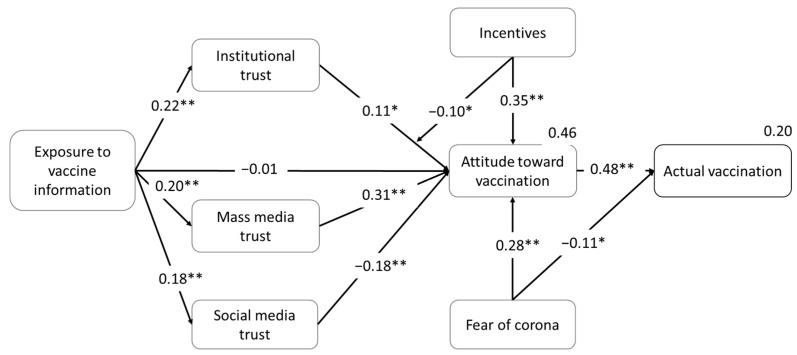
The path model. Path parameters are standardized parameter estimates. R^2^ are in the right corners. * *p* < 0.05; ** *p* < 0.01.

To test this study’s hypothesized relationships, a path analysis was conducted using structural equation modeling and Amos version 25 package. An interaction variable was added for the moderation of incentives. Acceptable levels of fit values (goodness of fit measures) were found, and the path model was valid (χ^2^ value (373) = 856.91, χ^2^/df < 3, *p* < 0.001; CFI = 0.955; NFI = 0.923; RMSEA = 0.052). The R squared for the endogenous variables indicates that the model accounts for 46% of the variance in attitude toward vaccination and 20% of the variance in actual vaccination. Table 3 presents test results of the conceptual model. The general path model, regression standardized coefficients, and their significance are shown in Figure 2.

Table 3 displays the variables’ direct relationships and their statistical measures. Figure 2 shows a direct positive relationship between attitude toward vaccination and actual vaccination (β = 0.48, *p* < 0.001). As expected, mass media trust and institutional trust have direct positive relationships with attitude toward vaccination (β = 0.31, *p* < 0.001; β = 0.11, *p* = 0.029, respectively). However, social media trust has a direct negative relationship with attitude toward vaccination (β = −0.18, *p* < 0.001). Therefore, H2, H4a and H5a were supported while H3a was not supported.

Additionally, the direct relationship between exposure to vaccine information and attitude is found to be insignificant (β = −0.01, *p* > 0.05), and significantly indirect (β = 0.06, bootstrap with 95% CI: 0.02–0.09; *p* = 0.012), where the relationship is mediated through its relationships with mass media trust, social media trust and institutional trust (β = 0.20, *p* < 0.001; β = 0.19, *p* < 0.001; β = 0.22, *p* < 0.001, respectively). Accordingly, H1 was not supported while H3b, H4b and H5b were merely generally supported.

The SEM statistics offer general results for the mediation effect. Therefore, we used Hayes’ PROCESS macro (model 4) with 5000 bootstrapped samples to check the specific mediation effect of mass media trust, social media, and institutional trust (fear of pandemic and vaccination status were added as covariates). The results indicate that the relationship between exposure to vaccine information and attitude was significantly mediated by each of the constructs: mass media trust (95% CI: = 0.013 to 0.098), social media (95% CI: = −0.058 to −0.006) and institutional trust (95% CI: = 0.002 to 0.064). These results support H3b, H4b, and H5b. Nevertheless, while mass media trust and institutional trust positively mediated this relationship, social media trust exhibits a negative mediation effect.

**Figure 3:**
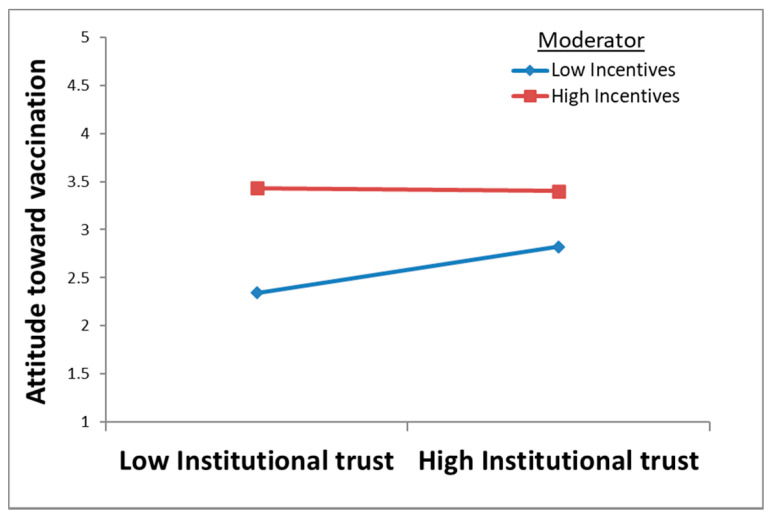
The moderation effect of incentives.

The effect of incentives on attitude toward vaccination is significantly positive (β = 0.35, *p* < 0.001). Furthermore, the regression results show that incentives negatively moderate the effect of institutional trust on attitude toward vaccination (β = −0.10, *p* = 0.014). This means that incentives dampen the positive relationship between institutional trust and attitude toward vaccination (see Figure 3). Hence, hypotheses H6 and H7 are supported.

Additionally, regarding the control variable, fear of pandemic has a positive relationship with attitude toward vaccination (β = 0.28, *p* < 0.001) and negative relationship with actual vaccination (β = −0.11, *p* = 0.015).

## 4. Discussion

In times of global pandemic such as COVID-19, worldwide institutions use communication campaigns to inform the public and convince them of the vaccine’s effectiveness. At the same time, these institutions are challenged by people’s hesitancy and reluctance to get vaccinated and the misleading information from anti-vaccination movements. Many institutions and organizations do not succeed with their campaigns due to a lack of knowledge about key factors in encouraging the undecided public. Vaccine hesitancy or refusal to get vaccinated is a complex issue involving individuals’ attitudes, beliefs and concerns, group influences, and contextual influences such as the novelty of the disease [4,5]. The present study aimed to understand the role of more distal factors involved in the persuasion process: trust in information sources, institutional trust, and incentives.

The current study demonstrates three major findings: first, the relationship between exposure to information about the vaccine and attitude toward vaccination is not direct as was expected from social learning theory [10]. The effect of exposure to information about COVID-19 vaccination on attitude formation was rather indirect and mediated by individuals’ trust of the players, such as trust in governmental institutions and media (mass media and social media). These results follow previous research demonstrating the essentiality of trust in public information judgment [25,60]. In times of pandemic, trust in information sources is vital for effectively delivering the message on the importance of vaccination and its acceptance [26,27].

Second, the effect of trust on attitude is source dependent. While the effect of mass media trust and institutional trust is positive, both as antecedents and moderators, the effect of social media was negative. This information positively influenced people who believe that mass media is credible in forming a positive attitude toward COVID-19 vaccination and institutional trust. However, people who believe that social media is a credible information source were negatively influenced toward COVID-19 vaccination and formed a negative attitude toward the vaccination. The false narratives can explain this, conspiracy theories and misinformation relating to COVID-19 vaccination, which are widely spread on social media platforms [1,34] and flourish out of control [35].

Third, the use of incentives [1] such as green pass documenting vaccination, monetary incentives, or vaccine accessibility (available in safe, familiar, and convenient places) can reduce the institutional mistrust effect and enhance vaccination attitude and actual vaccination. When there is a problem of trust, incentives are an effective tool to reduce the impact of mistrust and overcome hesitancy and refusal to be vaccinated [53,54]. Unlike coercive policies that may increase people’s resistance to vaccination [55], incentives give people a feeling of freedom with their decisions. Though incentives cannot overcome distrust, they can motivate distrusting individuals to make an effort to be vaccinated [56]. Still, the line between coercive policies and incentives such as the green pass can be quite blurred. While some may see the green pass positively as promoting the health of society and mutual social responsibility, others perceive it as a basis for discrimination and penalty for those who have not been vaccinated [61].

This study has some limitations that should be considered in future research. The unique situation in which the current study was conducted (i.e., where the vaccine supply exceeded its demand) constitutes its strength. Nevertheless, future research should check this study model in different countries to strengthen the results. Additionally, the current study was conducted at a specific time point and the data were collected from a representative sample of the entire population. This exploratory cross-sectional study indicates an association among the variables. Future research should conduct a case control study to support the causality among this study’s variables. Moreover, since the conditions in local markets differ across countries, more studies should be conducted, taking into account the specific regional and cultural conditions. Next, since the pandemic is evolving and dramatically changing, it is recommended to conduct additional studies at different times to detect any shifts in public attitudes and behavior. Finally, this study excluded age break 55+ since at the data collection phase this group (and not other medically vulnerable minorities) received a very dedicated treatment by the authorities. After focused communication campaigns, it was the first group to be vaccinated. They were also given direct access to the vaccinating teams all over the country. Hence, they could not contribute much to vaccine hesitancy. Other medically vulnerable minorities (such as those with chronic diseases) were not segregated from the general population and did not enjoy the same persuasion and accessibility efforts, therefore they were perceived as part of the general population and were included in the representative sample of the general population. Aside from medically vulnerable minorities other psychological and social determinants deserve focus in future research, such as level of education, conservative news consumption, political tendency, and conspiracy beliefs [62]. In addition, groups with specific ethnics origins that are associated with low vaccination uptake [63] and pregnant women [64] should be studied.

## 5. Conclusions

From a theoretical perspective, this study offers a conceptual framework that enhances our understanding of the effect of information sources and incentives on the persuasive process in health communication in times of pandemic such as COVID-19. It complements social learning theory by emphasizing the mediation effect of trust in the information sources and incentives on the learning process and consequent behavior.

This study offers some suggestions and preventive actions for public service managers and policy makers from a practical perspective. First, institutional trust and mistrust do not begin in the context of a crisis such as the current pandemic. Therefore, governmental authorities should develop a positive relationship with their community and cultivate a relationship of trust over time. These relationships will help effectively communicate and convince people during crises such as the COVID-19 pandemic. Additionally, when there are various levels of trust in the different governmental institutions, it is recommended to use those institutions with higher levels of trust as the official-governmental sources of information. Second, in light of the negative effect of social media on consumer willingness to be vaccinated, it is imperative to integrate trustworthy influencers on social media such as experts, e.g., doctors, researchers, and opinion leaders, to support the pro-vaccine information in order to balance the misinformation on this source. Moreover, people who were vaccinated can become agents of change and take part and assist in the persuasion process especially on social media, creating a new social norm. Additionally, this information should be continuously maintained to control media discourse to reach a higher share of voice. Third, after every round of vaccination, the authorities need to overcome hesitancy and refusal to be vaccinated by those who did not get vaccinated. This study indicates that incentives are suitable means to motivate this population. Accordingly, policy makers are advised to integrate common incentives in their vaccination promotion campaign, such as making vaccination available in safe, familiar, and convenient places, offering monetary incentives, and issuing green pass—which allows entrance to public places and cultural events. These incentives should be adjusted to the social and cultural environment of the targeted audience.

## Figures and Tables

**Table 1:** CFA—Item Factor Loading and Variable Reliability and Validity Measures.

Variables and Items	Std. Coef.	AVE	CR	Cronbach’s Alpha
**Attitude toward vaccination**		0.81	0.93	0.92
1. In general, vaccination against COVID-19 is a good thing	0.83 **			
2. It is better to be vaccinated against COVID-19 than to wait for herd immunity	0.95 **			
3. In my opinion being vaccinated against COVID-19 is better than not being vaccinated	0.92 **			
**Mass media trust**		0.88	0.97	0.97
1. The information I receive from media channels (T.V., radio, press or online news sits) is accurate	0.96 **			
2. The information I receive from media channels (T.V., radio, press or online news sits) is fair	0.96 **			
3. The information I receive from media channels (T.V., radio, press or online news sits) is reliable	0.96 **			
4. The information I receive from media channels (T.V., radio, press or online news sits) is unbiased	0.87 **			
**Social media trust**		0.84	0.95	0.95
1. The information I receive from friends and acquaintances on social network sites (Facebook, Instagram, Twitter) is accurate	0.95 **			
2. The information I receive from friends and acquaintances on social network sites (Facebook, Instagram, Twitter) is fair	0.93 **			
3. The information I receive from friends and acquaintances on social network sites (Facebook, Instagram, Twitter) is reliable	0.94 **			
4. The information I receive from friends and acquaintances on social network sites (Facebook, Instagram, Twitter) is unbiased	0.84 **			
**Institutional trust**		0.56	0.90	0.89
1. I trust the legal system in Israel	0.55 **			
2. I trust Israeli police service	0.72 **			
3. I trust the Israeli government	0.71 **			
4. I trust the local authorities	0.78 **			
5. I trust the Israeli health system	0.83 **			
6. I trust the medical insurance service	0.85 **			
7. I trust the military defense system	0.77 **			
**Exposure to vaccine information**		0.51	0.80	0.75
1. Level of exposure to information or talk about the COVID-19 vaccine on media channels (T.V., press, Internet)	0.74 **			
2. Level of exposure to information or talk about the COVID-19 vaccine on SNS (Facebook, Twitter, etc.)	0.56 **			
3. Level of exposure to information or talk about the COVID-19 vaccine on personal conversations with friends and family	0.70 **			
4. Level of exposure to information or talk about the COVID-19 vaccine on public space publications (street signs, city halls, etc.)	0.82 **			
**Incentives**		0.46	0.72	0.67
1. In my opinion, the incentives offered by the authorities to people who get vaccinated have much influence on their decision to be vaccinated	0.57 **			
2. In my opinion, the green pass (vaccinated certificate) is a strong motive for immunization	0.73 **			
3. In my opinion, if it is possible to be vaccinated in the workplace, or in shopping centers or in another accessible place—it will increase the willingness to be vaccinated	0.73 **			
**Fear of pandemic**		0.71	0.88	0.87
1. I still feel afraid due to the COVID-19 pandemic	0.79 **			
2. I am still afraid to go shopping outside my house because of the COVID-19 pandemic	0.92 **			
3. I still avoid doing many things out of fear of the COVID-19 pandemic	0.82 **			

** Standardized coefficients, *p* < 0.01; AVE = average variance extracted; CR = composite reliability.

**Table 2:** Correlations ^a^ between Variables and the maximum shared squared variance (MSV).

Variable	1	2	3	4	5	6	7	8
1. Actual vaccination	–	0.418 **	0.187 **	−0.035	0.138 **	0.089	0.174 **	0.083
2. Attitude toward vaccination	0.511	**0.81**	0.418 **	0.084	0.392 **	0.166 **	0.444 **	0.382 **
3. Mass media trust	0.090	0.175	**0.88**	0.525 **	0.552 **	0.187 **	0.300 **	0.219 **
4. Social media trust	0.001	0.007	0.276	**0.84**	0.335 **	0.161 **	0.180 **	0.090 *
5. Institutional trust	0.075	0.154	0.305	0.112	**0.56**	0.191 **	0.256 **	0.300 **
6. Exposure to vaccine inform.	0.020	0.028	0.035	0.026	0.036	**0.52**	0.150 **	0.238 **
7. Incentives	0.120	0.197	0.090	0.032	0.066	0.023	**0.46**	0.137 **
8. Fear of pandemic	0.084	0.146	0.048	0.008	0.090	0.057	0.019	**0.71**

Notes: *n* = 484; * *p* < 0.05, ** *p* < 0.01; ^a^ Correlations are in the upper right side while the MSV are in the lower left side; AVE are in bold diagonal.

**Table 3:** Direct and indirect variable relationships.

Relationship	Standardized Effect			Regression Weights (Direct)		
	Total	Direct	Indirect	Estimate	C.R.	*p*
Exposure to vaccine inform → Attitude toward vaccination	0.044	−0.011	0.055	−0.014	−0.239	>0.05
Attitude toward vaccination → Actual vaccination	0.477	0.477	0.000	0.168	9.890	<0.001
Mass media trust → Attitude toward vaccination	0.311	0.311	0.000	0.262	5.907	<0.001
Exposure to vaccine inform → Mass media trust	0.204	0.204	0.000	0.306	3.939	<0.001
Social media trust → Attitude toward vaccination	−0.176	−0.176	0.000	−0.153	−3.874	<0.001
Exposure to vaccine inform → Social media trust	0.185	0.185	0.000	0.269	3.577	<0.001
Institutional trust → Attitude toward vaccination	0.108	0.108	0.000	0.113	2.178	=0.029
Exposure to vaccine inform → Institutional trust	0.217	0.217	0.000	0.262	4.075	<0.001
Incentives → Attitude toward vaccination	0.353	0.353	0.000	0.417	6.160	<0.001
Incentives X Institutional trust → Attitude toward vaccination	−0.099	−0.099	0.000	−0.127	−2.466	=0.014
